# Microbiome Modulation in Acne Patients and Clinical Correlations

**DOI:** 10.3390/life14060688

**Published:** 2024-05-27

**Authors:** Marius-Anton Ionescu, Alin Laurentiu Tatu, Camelia Busila, Elena Roxana Axente, Nelly Badalato, Marc G. J. Feuilloley, Estelle Asquier, José Dario Martínez, Luc Lefeuvre

**Affiliations:** 1 Dermatology Department, University Hospital “Saint Louis”, University of Paris, 75010 Paris, France; dr.toni.ionescu@gmail.com; 2 Clinical Medical Department, Faculty of Medicine and Pharmacy, “Dunarea de Jos” University, 800008 Galati, Romania; 3 “Sfanta Cuvioasa Parascheva” Hospital of Infectious Diseases, 800179 Galati, Romania; 4 Multidisciplinary Integrated Center of Dermatological Interface Research MIC-DIR (Centrul Integrat Multidisciplinar de Cercetare de Interfata Dermatologica—CIM-CID), “Dunărea de Jos” University, 800201 Galati, Romania; 5 “Sf. Ioan” Emergency Clinical Paediatric Hospital, 800487 Galati, Romania; 6 Faculty of Medicine and Pharmacy, “Dunarea de Jos” University, 35 AI I Cuza St., 800010 Galati, Romania; elena.axente@ugal.ro; 7 GenoScreen, 59000 Lille, France; nelly.badalato@genoscreen.fr; 8 Research Unit UR4312 CBSA, University Rouen, 27000 Evreux, France; marc.feuilloley@univ-rouen.fr; 9 Laboratoires Dermatologiques d’Uriage, 92200 Neuilly sur-Seine, France; estelle.asquier@uriage.com (E.A.); luc.lefeuvre@uriage.com (L.L.); 10 Department of Internal Medicine, Faculty of Medicine, Hospital Universitario José Eleuterio González, Universidad Autónoma de Nuevo León, Monterrey 64460, Mexico; jose.martinezvil@uanl.edu.mx

**Keywords:** microbiota, skin, acne, emulsion

## Abstract

Background: The imbalance of skin microbiota in acne can induce changes leading to induction or to aggravation of chronic inflammatory lesions; complex mechanisms are involved. *Cutibacterium acnes* (*C. acnes)* ribotypes RT4 and RT5 express more biofilm and are associated with inflammatory acne lesions. *C. acnes* RT6 is a non-acne ribotype, beneficial for the skin. Objectives: In an open clinical trial, acne adults were included and assessed clinically at baseline and at month 2 using the Investigator Global Assessment of Acne (IGA) score. A topical emulsion was applied twice daily for 2 months (M2) in each included patient. In the same series of acne patients, skin swab samples were collected from acne patients at baseline and M2 from lesional and non-lesional skin; skin swabs were collected for the metagenomic long-read analysis of microbiota. Materials and Methods: Acne patients with a gravity score IGA of >1<3 were included in this pilot study. An emulsion of O/W formulated with vegetal extract of *Umbelliferae* associated with a polysaccharide at 1% was applied twice daily for 2 months. At baseline and M2 clinical assessments were made; skin swab samples were also taken for microbiota analysis from lesional and non-lesional skin in each included patient. Extractions of genomic DNA (gDNA) from swab samples from baseline and from M2 were made, followed by full-length (V1–V9) amplification of the 16S rDNA and sequencing of amplicon libraries for strain-level bacterial community profiling. Results: In a series of 32 adult acne patients, the mean initial IGA scale was 3.1; at M2 the IGA scale was 1.5 (*p* < 0.001). The mean decrease in acne lesions was by 63%. Microbiome metagenomic long-read analysis in these series was mainly dominated by *C. acnes* followed by *Staphylococcus epidermidis* (*S. epidermidis)*. The density of *C. acnes* ribotypes RT6 (non-acne strain) was increased at M2 compared to baseline and the density of ribotypes *C. acnes* RT1 to RT5 was decreased at M2, compared to baseline (*p* < 0.0001). *S. epidermidis* ribotypes (1 to 36) were non significantly increased at M2, compared to baseline (*p* < 0.1). Conclusions: In a series of 32 acne patients that applied an emulsion based on vegetal extract of *Umbelliferae* and a polysaccharide at 1% twice daily, a significant clinical improvement in IGA scale for acne lesions was seen at M2, compared to baseline (*p* < 0.0001). The clinical improvement was correlated with an improvement in skin microbiome at M2 compared to baseline, indicated by the increase in the relative abundance of non-acne strain of *C. acnes* ribotype 6 and of the decrease in the relative abundance of acne strains ribotypes *C. acnes* RT1 to RT5.

## 1. Introduction

**Figure 1:**
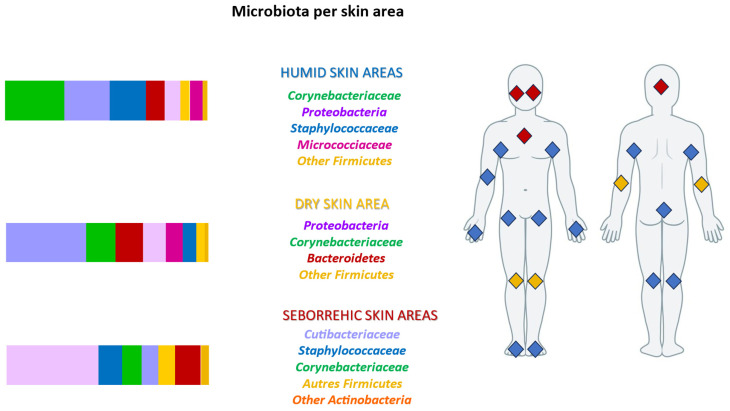
Variability of the microbiota according to the skin regions: prevalence of *Cutibacterium* in the modified seborrhea zone [1].

The skin microbiota (SM), defined as the micro-organisms living on the skin surface, is the second biggest microbiota of humans, after the intestinal microbiota. The micro-organisms (bacteria, yeasts, archaea, and acarian) and viruses are distributed on the skin surface and in the skin’s adnexa (sweat and sebaceous glands). The adult SM bacteria are composed of four main *phyla* (*Actinobacteria*, *Firmicutes*, *Proteobacteria,* and *Bacteroidetes*) and three predominant *genus* (*Corynebacterium*, *Staphylococcus,* and *Cutibacterium*). The distribution of SM varies according to the regions *of the human body* and depending on skin micro-environments (dry or humid or oily skin). The genus *Cutibacterium* is predominant in seborrheic skin areas (Figure 1). The pilosebaceous follicle is a micro-aerobic environment, rich in lipids, adapted to the survival of *Cutibacterium acnes* (CA), Gram-positive, anaerobic bacteria.

If the quantity of CA is the same on acne and non-acne skin, the difference concerns the loss of diversity of CA *phylotypes*, with the predominance of the IA1 phylotype in inflammatory acne lesions—this particular phylotype is more pathogenic and produced more biofilm. The *C.acnes* ribotype RT6 is commensal and non-acnegenic.

Metagenomic, full-length, V1–V9 sequencing of 16sRNA precisely analyzed the different CA ribotypes and their relative abundance. The distribution of ribotypes varies in the pilosebaceous follicle in acne lesions, due to several mechanisms, specific receptors’ activation, and also due to biofilm formation by CA RT4 and RT5. Thus, two strains of CA were selected to be studied: CA phylotype IA1—ribotype 4 (RT4), acne pathogen, pro-inflammatory; and CA phylotype II—ribotype 6 (RT6), non-acnegenic commensal. CA RT4 grows and multiplies easier in a lipid, anaerobic environment, where is induces more biofilm formation and pro-inflammatory enzymes, as opposed to CA RT6—non-acne ribotype, beneficial for the skin.

In the pilosebaceous follicle, as on the skin surface, bacterial biofilms are mixed and made of several bacterial *phylotypes* [2]. These *phylotypes*—individualized, mixed, as endosymbionts, or present with their different biofilms—interact permanently with the skin, producing different enzymes and inducing host cell’ reactions [3,4].

The pathophysiology of acne is complex and multifactorial [5,6]:

- Modifications of the microbiome: loss of the diversity of CA phylotypes, predominance of CA RT4 and RT5, ribotypes produce more biofilm and become more adherent to skin cells, formation of the biofilm increases bacterial virulence, antibiotic resistance, secretion of exo-enzymes, and prolonged bacterial survival [4,5];
- Inflammatory factors: activation of innate immunity (Toll-Like Receptors, inflammasome, IL-1, and caspase) and Th17 pathway activation with the expression of IL-17A/F [6];
- Factors linked to the pilosebaceous follicle: genetic predisposition to hyperseborrhea, hyperkeratinisation, an increased sensitivity of receptors to androgens, activation of neuroreceptors of neuromediators such as substance P, etc.;
- Environmental factors that are part of the exposome.

Besides conventional therapies of acne (e.g., topical retinoids, benzoyl peroxide, oral cyclins, retinoids, etc.), new targets in acne have been identified (Table 1).

### Objectives

In an open, multicentric clinical study on adult acne patients we sought to achieve the following: A. to assess the effects on acne lesions of a topical O/W emulsion formulated with a vegetal extract of *Umbelliferae* and a polysaccharide at 1%, (E1), applied twice daily for 2 months; B. to analyze the skin microbiota of lesional and non-lesional skin in the same series of patients.

## 2. Materials and Methods

**Figure 2:**
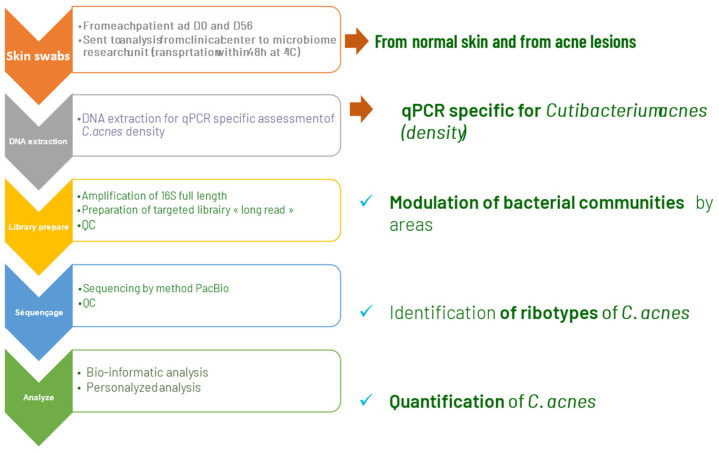
Protocol of metagenomic *long-read* study of microbiome in acne patients treated using emulsion E1.

**Figure 3:**
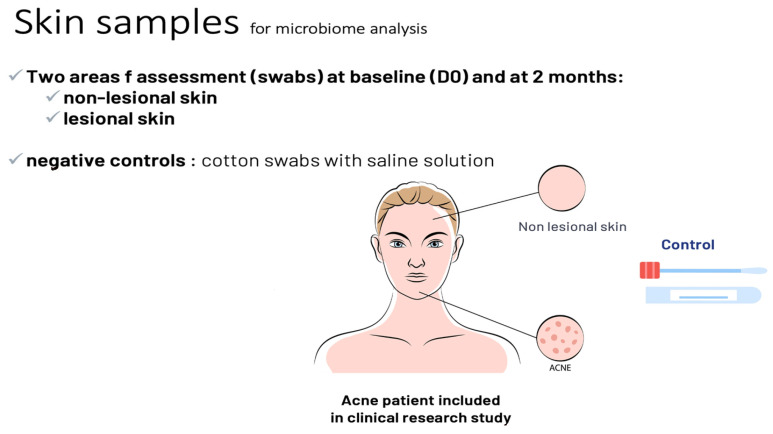
Protocol (part 2) of metagenomic long-read study of microbiome in acne patients treated using emulsion E1.

In an open clinical trial, we included adult acne patients (over 18 years old) with facial acne, grade 1 to 3 on the scale Investigator Global Assessment of Acne (IGA) [7]. An oil-in-water emulsion (O/W formulated with a purified vegetal extract of *Umbelliferae* and a polysaccharide at 1%), was applied twice daily to the whole facial skin area, on lesional and non-lesional skin areas. Clinical assessments, lesion count, IGA score, and clinical photographs (Visia^TM^) were made and taken at baseline, one month (M1), and at two months (M2) by the investigating dermatologists. A microbiota assessment was made using skin swabs taken at baseline and M2 from lesional and non-lesional skin of each patient: the skin workflow (GenoBiome^®^ Skin, GenoScreen, Lille, France) consisted of long-read V1–V9 16S rDNA metagenomic and *C. acnes* quantitative PCR; for a strain-level analysis of the skin microbiota, *C. acnes* and *S. epidermidis* ribotyping and quantitative measurement were conducted. The workflow was made of 4 steps: a. extraction of genomic DNA (gDNA) from swab samples; b. amplification of the full-length (V1–V9) of the 16S rDNA; c. sequencing of amplicon libraries on a single PacBio^®^ Sequel II; and d. bioinformatic analyses for strain-level bacterial community profiling. Samples were received in batches. Upon reception, samples were immediately pre-processed, and a control (molecular grade water) was added for each batch. Upon reception of the last batch, all samples were processed for gDNA extraction. The V1–V9 16S rDNA amplicons and libraries were prepared following an optimized PacBio^®^ protocol. The final library, an equimolar pool of libraries (samples from the study and a total of 8 quality controls), was quality controlled using capillary electrophoresis. Sequencing was performed on a PacBio Sequel II platform. Sequencing data were processed through an optimized long-read 16S rDNA pipeline based on QIIME2. The taxonomic profiling was made by grouping unique sequences of Amplicon Sequence Variants (ASVs), which were then assigned to taxa using a curated, well-established public database to obtain a strain-level of bacterial community profiling with annotation down to the ribotypes level (Figure 2 and Figure 3). Wilcoxon tests were performed on counts for all samples assigned to any *Cutibacterium* or *Staphylococcus* ribotypes.

## 3. Results

**Figure 4:**
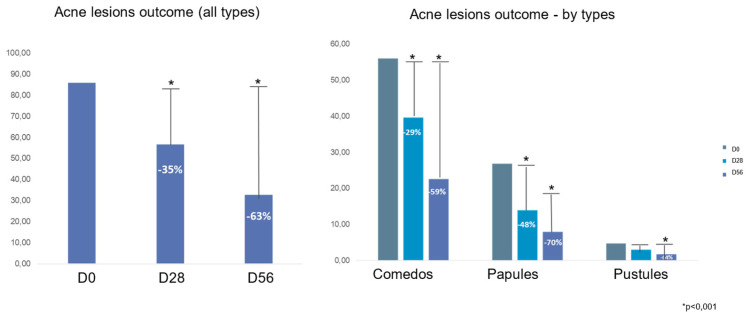
Acne lesion outcomes in patients treated using the emulsion E1.

**Figure 5:**
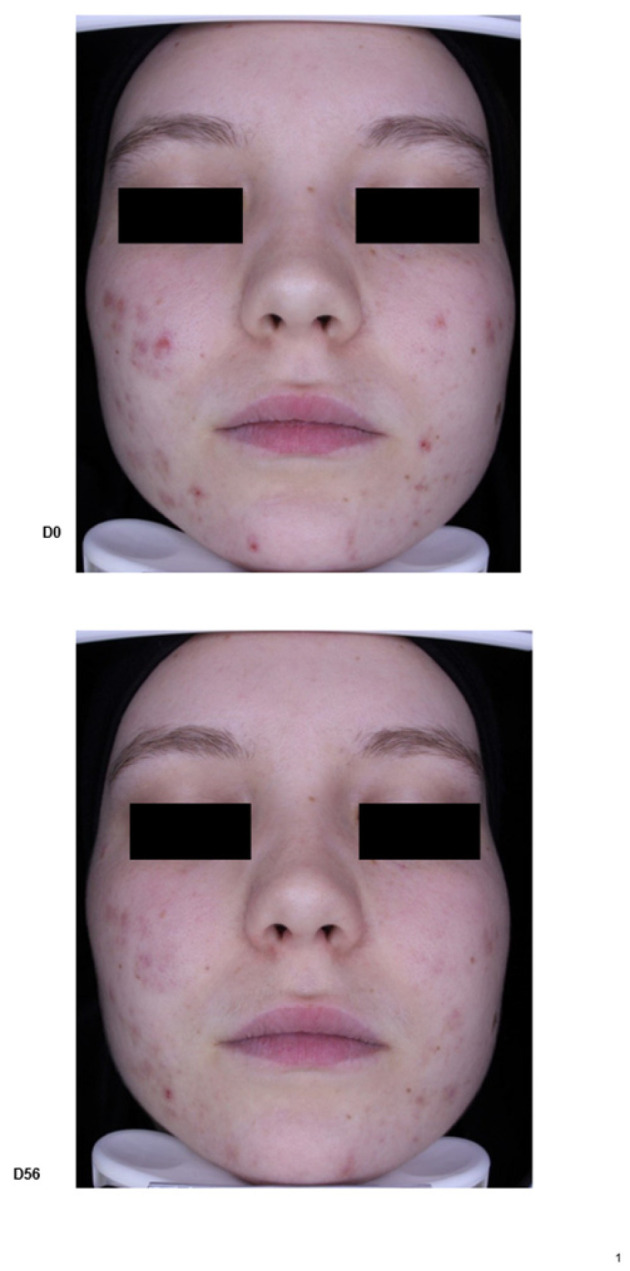
Patient treated using emulsion E1, applied *twice daily* for 2 months (baseline D0, M2—D56).

A. Clinical trial: Thirty-two patients were included in this pilot trial. Analysis of the results showed a significant decrease in the IGA score to 1.5 at M2 compared to 3.1 at baseline (*p* < 0.001); the mean decrease in acne lesions was by −63% at M2 compared to baseline (*p* < 0.001). At M2, we noticed a significant reduction in comedones and microcysts by −59% compared to baseline (*p* < 0.001) and a decrease in papules by −70% and pustules by −64% at M2 compared to baseline (*p* < 0.001) (Figure 4 and Figure 5).

**Figure 6:**
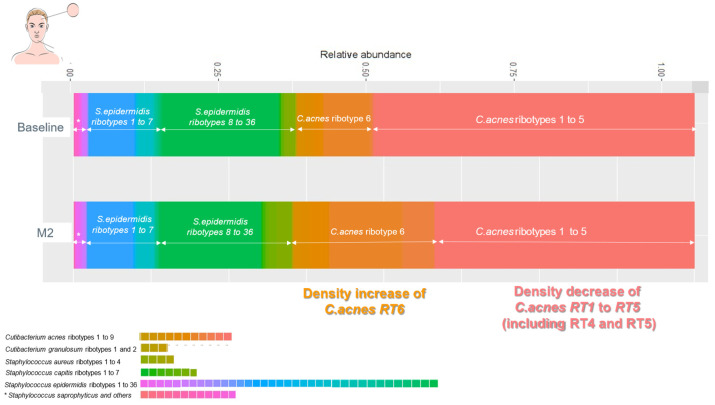
Evolution of skin microbiome in non-lesional skin before and after application of emulsion E1 for 2 months.

**Figure 7:**
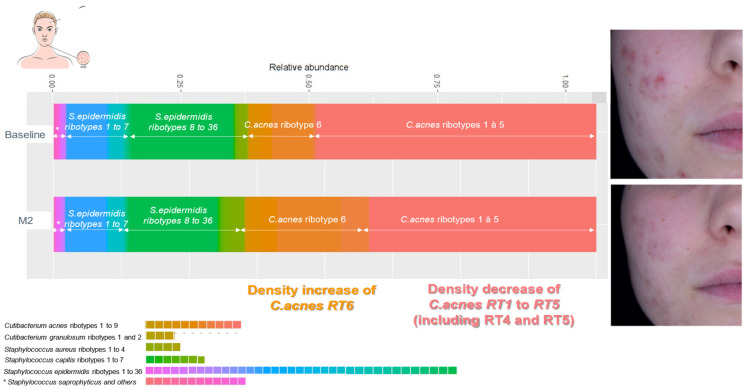
Evolution of skin microbiome in lesional skin before and after application of emulsion E1 for 56 days.

B. The metagenomic long-read study of acne patients showed a skin microbiota dominated, in lesional and non-lesional skin, by *C. acnes* followed by *S. epidermidis*. The relative abundance of non-acne strain *C. acnes* ribotype 6 increased at M2 compared to baseline; *a* more important increase inf relative abundance of *C. acnes* RT6 was seen in lesional skin (*p* < 0.0001). The relative abundance of pro-inflammatory acne strains *C. acnes* ribotypes 4 and 5 decreased at M2 compared to baseline in both lesional and non-lesional skin (*p* > 0.0001). *S. epidermidis* ribotypes (1 to 36) non-significantly increased at M2 compared to baseline (*p* < 0.1) (Figure 6 and Figure 7).

## 4. Discussion

There are several instruments for measuring acne severity that are not interchangeable because they do not measure the same disease components [8,9]. Modern lifestyles involving exposure to skin care products, cosmetics, medications, and pollution induce changes in the skin microbiome [10,11,12,13,14]. Acne is a multifactorial disease that can be improved by daily use of appropriate skin care products. In older adults, the relative abundance of different phylae within the skin microbiome has been reported, representing a risk for antimicrobial resistance and nosocomial strains, with the risk of dissemination of multidrug-resistant pathogens [15,16,17]. Skin aging is also linked to changes in the skin microbiome and is significantly influenced by the urban environment and lifestyle.

The commensal bacterium *Cutibacterium acnes*—*C. acnes* (previously *Propionibacterium acnes—P. acnes*) is part of the commensal microbiota, but it can evolve as an opportunistic pathogen in acne vulgaris, mainly pro-inflammatory ribotypes RT4 and RT5. These ribotypes belonging to phylotype IA1 are more virulent and more resistant to topical and systemic treatments of acne [18,19,20].

In a series of 101 volunteers (n = 49 acne patients and n = 52 healthy individuals), the skin microbiome of each participant was compared via metagenomic analysis, focusing *Propionibacterium acnes (today reclassified as Cutibacterium acnes—C. acnes)*. The relative abundance of *C. acnes* was similar in acne individuals; the long-read analysis showed that the ribotypes (RT) of *C. acnes* (n = 10 ribotypes) were significantly different in the two groups. RT4 and RT5 were significantly associated with acne; the relative abundance of RT6 was significantly higher in the skin of healthy individuals [21].

A recent update was published on current acne treatments (benzoyl peroxide, oral isotretinoin, and antibiotics) that may affect the skin microbiome in acne patients. The imbalance of microbiota is also accentuated by the risk of antimicrobial resistance. The role of non-antibiotic acne treatments is underlined [22].

*Staphylococcus epidermidis* (*S. epidermidis*) is a saprophyte bacteria (opportunistic pathogen) that has an “interactome type” of interaction *with C. acnes*, modulating functional microbiota diversity. There are species that can protect against colonization by skin pathogens and modulate the immune system [23,24,25]. Species interactions could compromise the balance of the skin microbiota, with *S. epidermidis* having an arsenal of mechanisms that can inhibit *C. acnes* [26]. The influence of environmental factors, such as low-glycemic-index foods, should be taken into account in the management of acne [27].

## 5. Conclusions

In a series of 32 acne patients that applied, twice daily, an emulsion based on vegetal extract of *Umbelliferae* and a polysaccharide at 1%, a significant clinical improvement in the IGA scale of acne lesions was seen at M2, compared to baseline (*p* < 0.0001). The clinical improvement was correlated with the improvement in the skin microbiome at month 2 compared to baseline, as indicated by the increase in the relative abundance of the non-acne strain of *C. acnes ribotype 6* and the decrease in the relative abundance of acne strains *ribotypes C. acnes RT1 to RT5* (*p* < 0.0001).

## Figures and Tables

**Table 1:** New targets in acne treatment.

Microbiome	Pro- and pre-biotics antimicrobial peptides (post-biotics) bacteriophages targeting *C. acnes*
Innate immunity	Vaccination (anti-CAMPs)
*C. acnes*	Inhibitors of biofilm of *C. acnes* phylotype IA1—ribotypes RT4 and RT5 [4,5].
Stress catecholamines	Epinephrine and norepinephrine [6].

## Data Availability

The original contributions presented in the study are included in the article, further inquiries can be directed to the corresponding author.

## Abbreviations

Investigator Global Assessment of Acne	IGA
Month 2	M2
Genomic DNA	gDNA
Skin microbiota	SM
*Cutibacterium acnes*	*C. acnes* (CA)
Ribotype 4	RT4
Ribotype 6	RT6
Amplicon Sequence Variants	ASVs
